# The Flavonoid Apigenin Downregulates CDK1 by Directly Targeting Ribosomal Protein S9

**DOI:** 10.1371/journal.pone.0073219

**Published:** 2013-08-29

**Authors:** Yosuke Iizumi, Masakatsu Oishi, Tomoyuki Taniguchi, Wakana Goi, Yoshihiro Sowa, Toshiyuki Sakai

**Affiliations:** 1 Department of Molecular-Targeting Cancer Prevention, Graduate School of Medical Science, Kyoto Prefectural University of Medicine, Kawaramachi-Hirokoji, Kamigyo-ku, Kyoto, Japan; 2 Department of Urology, Graduate School of Medical Science, Kyoto Prefectural University of Medicine, Kawaramachi-Hirokoji, Kamigyo-ku, Kyoto, Japan; University of Hawaii Cancer Center, United States of America

## Abstract

Flavonoids have been reported to inhibit tumor growth by causing cell cycle arrest. However, little is known about the direct targets of flavonoids in tumor growth inhibition. In the present study, we developed a novel method using magnetic FG beads to purify flavonoid-binding proteins, and identified ribosomal protein S9 (RPS9) as a binding partner of the flavonoid apigenin. Similar to treatment with apigenin, knockdown of RPS9 inhibited the growth of human colon cancer cells at the G2/M phase by downregulating cyclin-dependent kinase 1 (CDK1) expression at the promoter level. Furthermore, knockdown of RPS9 suppressed G2/M arrest caused by apigenin. These results suggest that apigenin induces G2/M arrest at least partially by directly binding and inhibiting RPS9 which enhances CDK1 expression. We therefore raise the possibility that identification of the direct targets of flavonoids may contribute to the discovery of novel molecular mechanisms governing tumor growth.

## Introduction

Flavonoids in vegetables and fruits have antitumor properties. Flavonoids were originally considered to exert anticarcinogenic effects through modification of cell surface signal transduction [1,2]. In contrast, we have first demonstrated that a typical flavonoid quercetin causes cell cycle arrest at the G1 phase [3]. After that, we and others have found that many other flavonoids cause cell cycle arrest at the G1 or G2/M phase by regulating a variety of cell cycle regulators [4–12]. However, little is known about the direct targets of flavonoids and their precise growth-inhibitory mechanisms.

The ribosome is composed of many ribosomal proteins and performs protein synthesis. However, ribosomal proteins are known to have other functions, which are called extraribosomal functions [13,14]. For instance, ribosomal proteins L5, L11, and L23 directly bind and inhibit murine double minute-2 (MDM2), leading to p53 activation [15–17]. Ribosomal protein S3 (RPS3) is a subunit in nuclear factor-κB (NF-κB) complexes [18], and the enterohemorrhagic *E. coli* O157:H7 effector NleH inhibits RPS3, resulting in suppression of the NF-κB pathway [19,20]. Although flavonoids activate p53 [21] and inhibit the NF-κB pathway [22], the relationship between flavonoids and these extraribosomal functions is unknown.

To elucidate the precise molecular mechanisms of tumor growth inhibition by flavonoids, we attempted to identify the binding proteins of a major flavonoid apigenin using magnetic FG beads, which have succeeded in determining the receptors of pharmacological agents such as thalidomide [23,24].

## Materials and Methods

### Reagents

Apigenin was purchased from Wako Pure Chemical Industries (Osaka, Japan) and dissolved in DMSO. Anti-ribosomal protein S9 (Abcam, Cambridge, UK) and p21 (Santa Cruz Biotechnology Inc., Santa Cruz, CA, USA) rabbit polyclonal antibodies, and anti- cyclin-dependent kinase 1 (CDK1) (Santa Cruz), cyclin B1, histidine-tag, ribosomal protein S6 (Cell Signaling Technology, Danvers, MA, USA), and β-actin (Sigma, St. Louis, MO, USA) mouse monoclonal antibodies were used as primary antibodies.

### Cell culture

Human colon cancer cell lines HT-29 and SW620 were obtained as cell lines of the NCI-60 from the National Cancer Institute Developmental Therapeutics Program (NCI DTP). HT-29 and SW620 cells were cultured in DMEM supplemented with 10% fetal bovine serum, 4 mM glutamine, 50 units/ml penicillin, and 100 µg/ml streptomycin at 37°C in 5% CO_2_.

### Cell viability assay

Cell viability was determined using the Cell Counting Kit-8 (CCK-8) (Dojindo, Kumamoto, Japan). CCK-8 solution was added to the medium of apigenin-treated or siRNA-transfected HT-29 cells. Absorbance of the samples (450 nm) was measured using a multi-plate reader (Viento, Dainippon, Osaka, Japan) after 4 hr of incubation.

### Cell cycle analysis

HT-29 cells were treated with apigenin for 24 hr or transfected with siRPS9 or siCtrl and incubated for 72 hr. The cells were then harvested by trypsinization. After centrifugation, the cells were suspended in PBS containing 0.1% Triton X-100, 150 µg/ml RNase A, and 50 µg/ml propidium iodide to prepare and stain nuclei. The suspension was filtered through nylon mesh (Kurabo, Osaka, Japan). DNA content in stained nuclei was analyzed by FACSCalibur (Becton Dickinson, Franklin Lakes, NJ, USA).

### Immunoblot analysis

HT-29 and SW620 cells treated with apigenin or siRNAs were lysed with RIPA buffer (50 mM Tris-HCl [pH 8.0], 150 mM NaCl, 1% NP-40, 0.5% deoxycholic acid, 0.1% SDS, 1 mM DTT, 0.5 mM PMSF) for 30 min at 4°C and centrifuged. The supernatants were subjected to SDS-PAGE, and analyzed by immunoblotting.

### Preparation of apigenin-fixed beads

Magnetic FG beads with epoxy linkers were purchased from Tamagawa Seiki (Nagano, Japan). The beads were mixed with apigenin in DMF containing potassium carbonate at 37°C for 24 hr, washed twice with DMF, and then twice with deionized water. The resulting beads were stored at 4°C.

### Purification and identification of apigenin-binding proteins with apigenin-fixed beads and MALDI-TOF MS

HT-29 cells were lysed with binding buffer (50 mM Tris-HCl [pH 8.0], 150 mM NaCl, 1% NP-40, 1 mM DTT, 0.5 mM PMSF) for 30 min at 4°C and centrifuged. The supernatants were used as whole cell extracts of HT-29 cells. The extracts were incubated with apigenin-fixed or empty beads for 4 hr at 4°C. The beads were washed three times with binding buffer. The bound proteins were eluted with Laemmli dye, subjected to SDS-PAGE, silver-stained, and subjected to in-gel digestion by Sequencing Grade Modified Trypsin (Promega, Madison, WI, USA). The peptide fragments were analyzed using an Autoflex II mass spectrometer (Bruker Daltonics, Billerica, MA, USA). For the competitive binding assay, HT-29 extracts were incubated with free apigenin for 1 hr before incubation with the beads.

### Plasmid construction and preparation of recombinant proteins

The 245-bp region between -245 and -1 of the CDK1 promoter was subcloned from the human genome into the pGV-B2 luciferase assay vector (Toyo Ink, Tokyo, Japan). The cDNA encoding RPS9 (accession #NM_001013) was subcloned from a HT-29 cell cDNA library into pET-14b (Novagen, Madison, WI, USA). Recombinant histidine-tagged RPS9 protein was expressed in the *E. coli* strain BL21-CodonPlus (DE3)-RIPL (Stratagene, La Jolla, CA, USA) and purified using Ni-NTA Agarose (QIAGEN, Hilden, Germany).

### RNAi

The following Stealth RNAi oligonucleotides (Invitrogen, Carlsbad, CA, USA) were used: siRPS9 #1, 5’-CAUACUCGCCGAUCAGCUUCAGCUC-3’; siRPS9 #2, 5’-AUGUAAUCCAGCUUCAUCUUGCCCU-3’; siRPS6 #1, 5’-AAAGUUUGCGGAUUCUGCUAGCUCU-3’; siRPS6 #2, 5’-ACUGGCGGACAUCAUCUUCUUUAGA-3’. Only sense strands are shown. A Stealth RNAi negative control (siCtrl) (Invitrogen) was used. Transfection of siRNA oligos was performed using Lipofectamine RNAiMAX (Invitrogen).

### Real-time RT-PCR

Total cellular RNA was extracted from HT-29 cells using Sepasol-RNA I super (Nacalai Tesque, Kyoto, Japan), and cDNA was synthesized from total RNA using High-Capacity cDNA Reverse Transcription Kits (Applied Biosystems, Melbourne, Australia). cDNA was amplified by PCR using TaqMan Probes (Applied Biosystems) and an ABI 7300 Real-time PCR System (Applied Biosystems).

### Luciferase assay

The CDK1 reporter plasmid (pCDK1PF) or pGV-B2 (an empty plasmid) was transfected into HT-29 cells using Lipofectamine 2000 (Invitrogen). After 8 hr, the cells were treated with apigenin for 16 hr and then lysed. Luciferase activities of the cell lysates were measured using Luciferase Assay Reagent (Promega) and a Lumat LB 9507 luminometer (Berthold Technologies, Bad Wildbad, Germany) and normalized to the amounts of total protein in the cell lysates.

### Detection of newly synthesized proteins

HT-29 cells were treated with apigenin for 24 hr and incubated in methionine-free DMEM containing apigenin for 1 hr. The cells were then treated with 50 µM L-azidohomoalanine (Invitrogen) for 1 hr and lysed with the lysis buffer (50 mM Tris-HCl [pH 8.0], 1% SDS, 1× cOmplete, Mini, EDTA-free (Roche Diagnostics, Indianapolis, IN, USA), 250 units/ml Benzonase (Novagen)). The proteins containing L-azidohomoalanine in the lysates were biotinylated with Click-iT Biotin Protein Analysis Detection Kit (Invitrogen). The biotinylated proteins were detected by immunoblotting with Pierce High Sensitivity Streptavidin-HRP (Thermo Scientific, Waltham, MA, USA).

### Statistics

Data are represented as means and standard deviation (SD). All experiments were performed in triplicate. Comparisons were performed using one-way ANOVA followed by Bonferroni *post-hoc* tests or unpaired Student’s *t*-test.

## Results and Discussion

### Apigenin induces G2/M arrest with downregulation of CDK1

**Figure 1:**
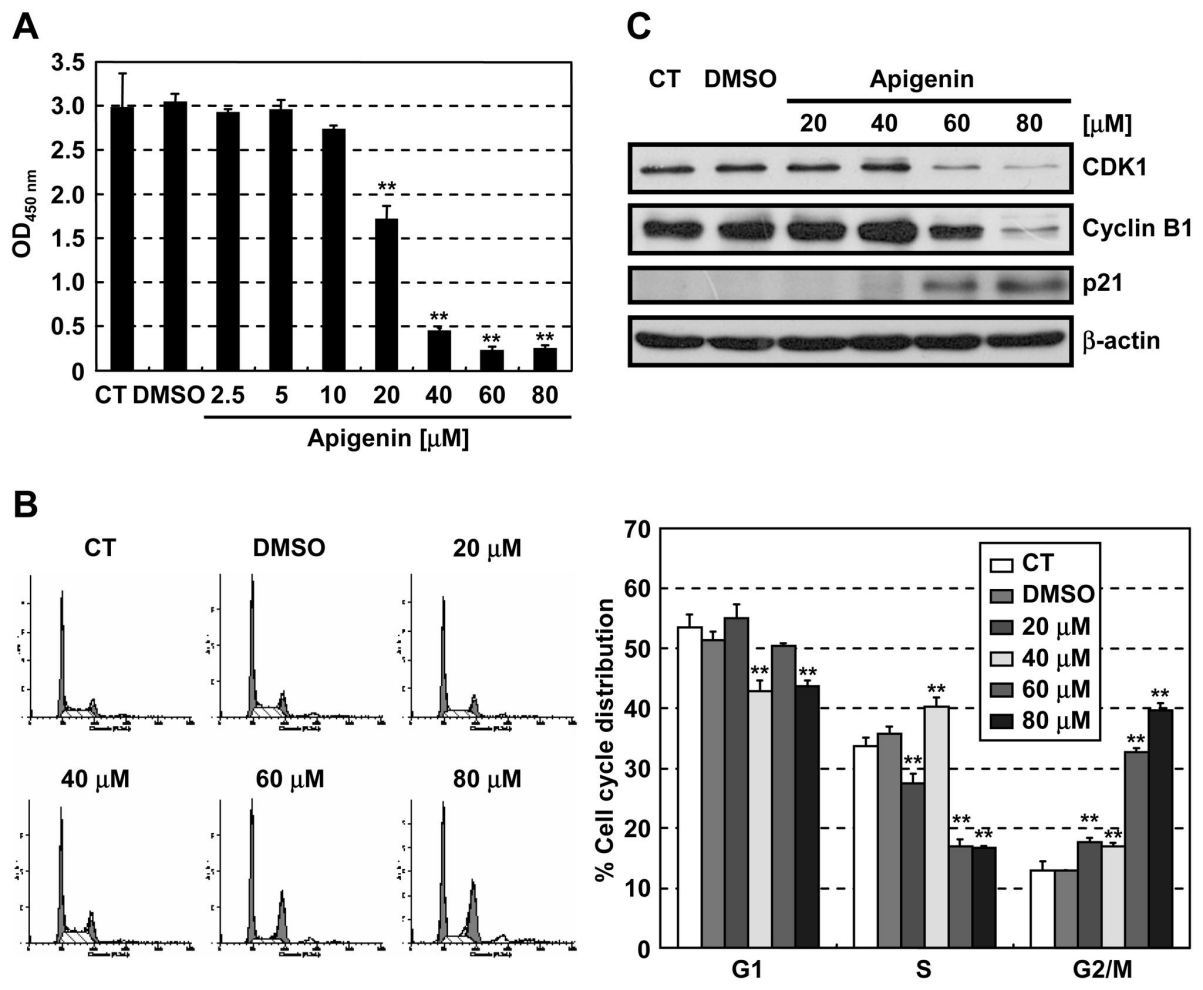
Apigenin induces cell cycle arrest at the G2/M phase. HT-29 cells were treated with the indicated concentrations of apigenin. (A) Relative viability of cells treated with apigenin for 72 hr was measured using the Cell Counting Kit-8. (B) Cell cycle analysis of cells treated with apigenin for 24 hr using flow cytometry. (C) Immunoblot analysis of G2/M phase regulators in cells treated with apigenin for 24 hr. CT: control, Data are means ± SD (n = 3). **P < 0.01 relative to control.

Apigenin, one of the major flavonoids, is contained in various vegetables and fruits. Apigenin has anticarcinogenic and antitumor activities *in vivo* [25–27] and causes cell cycle arrest at the G2/M phase in many kinds of cancer cells [5,28–30] by upregulating the CDK inhibitor p21 [31,32] and downregulating CDK1 and cyclin B1 [29]. In the present study, we also found that apigenin inhibited the growth of human colon cancer HT-29 cells in a dose-dependent manner (Figure 1A), and induced cell cycle arrest at the G2/M phase (Figure 1B). The G2/M phase arrest was associated with downregulation of CDK1 and cyclin B1, and upregulation of p21 (Figure 1C).

### Identification of ribosomal protein S9 as an apigenin-binding protein

**Figure 2:**
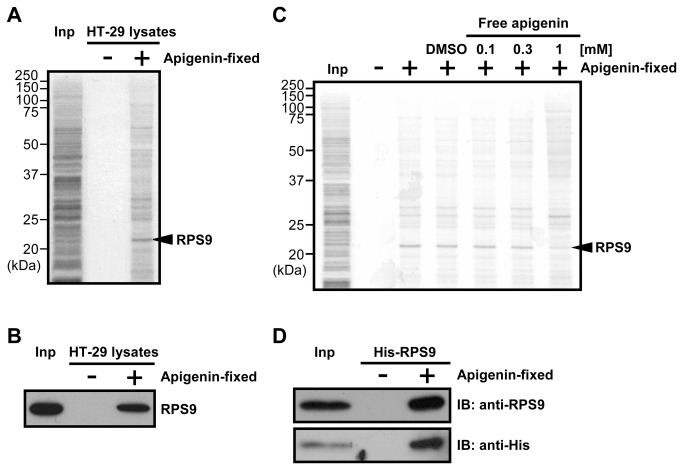
Apigenin directly binds to RPS9. (A) Apigenin-binding proteins were purified from whole cell extracts of HT-29 cells with apigenin-fixed (+) or empty (-) beads, and detected by silver staining. Mass spectrometry analysis identified RPS9 as an apigenin-binding protein. Inp: Whole cell extracts of HT-29 cells. (B) Confirmation of mass spectrometry analysis by immunoblotting with an anti-RPS9 antibody. Inp: Whole cell extracts of HT-29 cells. (C) Competition binding assay. The indicated concentrations of free apigenin were added to HT-29 cell extracts. After 1 hr, the extracts were incubated with apigenin-fixed (+) or empty (-) beads. Apigenin-binding proteins were purified and detected by silver staining. Inp: Whole cell extracts of HT-29 cells. (D) Purified recombinant His-RPS9 was incubated with apigenin-fixed (+) or empty (-) beads, and bound His-RPS9 was detected by immunoblotting with anti-RPS9 and anti-His antibodies. Inp: Purified recombinant His-RPS9.

To identify the direct target of apigenin, apigenin-binding proteins were purified using magnetic FG beads with epoxy linkers [23,24]. A system was developed in which flavonoids were conjugated onto the beads with potassium carbonate. Phenolic hydroxyl groups of flavonoids covalently bind to epoxy groups of the beads in the system. Apigenin was covalently conjugated to the beads using this system (Figure S1), and apigenin-fixed beads were incubated with whole cell extracts of HT-29 cells. One major apigenin-binding protein was purified from HT-29 cell extracts. This protein was identified as ribosomal protein S9 (RPS9) by mass spectrometry (Figure 2A). Identification of this protein was confirmed by immunoblotting (Figure 2B). Binding of RPS9 was selectively competed with increasing concentrations of apigenin (Figure 2C), suggesting that RPS9 specifically interacted with apigenin. Purified recombinant histidine-tagged RPS9 (His-RPS9) also bound to apigenin-fixed beads, suggesting that this interaction was direct (Figure 2D). Since RPS9 is an RNA-binding protein, we further examined whether RNA was required for this interaction. As shown in Figure S2, apigenin bound to RPS9 in the presence of RNase A. These results indicate that apigenin directly binds to RPS9.

### Knockdown of RPS9 induces G2/M arrest by downregulating CDK1

**Figure 3:**
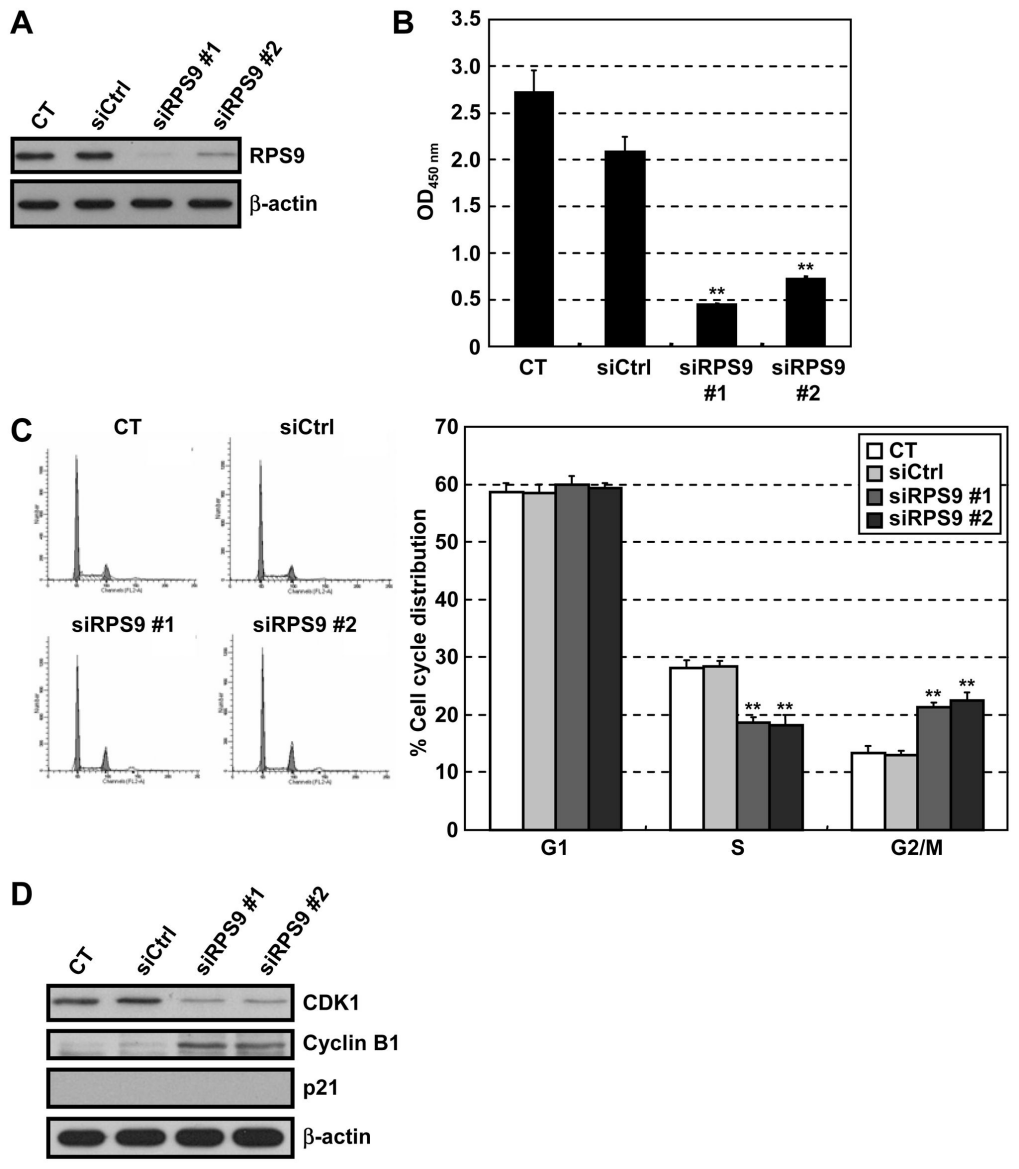
Knockdown of RPS9 causes cell cycle arrest at the G2/M phase by repressing CDK1 expression. HT-29 cells were transfected with two different siRNAs targeting human RPS9 (siRPS9 #1 and #2) or a non-targeting siRNA (siCtrl), and incubated for 72 hr. (A) Immunoblot analysis using an anti-RPS9 antibody. β-actin serves as a loading control. (B) Relative viability of transfected cells was examined using the Cell Counting Kit-8. (C) Cell cycle analysis of transfected cells using flow cytometry. (D) Immunoblot analysis of G2/M phase regulators in transfected cells. CT: control, Data are means ± SD (n = 3). **P < 0.01 relative to siCtrl.

Recently, several extraribosomal functions of ribosomal proteins have been discovered, such as regulation of the stability of the p53 tumor-suppressor gene product [13,14]. Knockdown of RPS9 induced the expression of p53, and inhibited cell growth at the G1 phase in human osteosarcoma U2OS cells, which express wild-type p53 [33,34]. The effect of knockdown of RPS9 on the growth of HT-29 cells, which express mutant p53, was therefore examined next. The growth of HT-29 cells was suppressed by siRNA-mediated depletion of RPS9 (Figure 3A and B). This growth inhibition was caused by cell cycle arrest at the G2/M phase (Figure 3C). The G2/M phase arrest was associated with the downregulation of CDK1 (Figure 3D). In contrast, cyclin B1 was upregulated in RPS9-knockdown cells, perhaps because cyclin B is expressed at maximum level in the G2/M phase [35]. On the other hand, knockdown of ribosomal protein S6 (RPS6) also downregulated CDK1 (Figure S3), suggesting that CDK1 downregulation may be a common cellular response due to loss of a ribosomal protein. These results suggest that knockdown of RPS9 arrests the cell cycle at the G2/M phase by downregulating CDK1 in a p53-independent manner.

### Knockdown of RPS9 downregulates CDK1 at the promoter level

**Figure 4:**
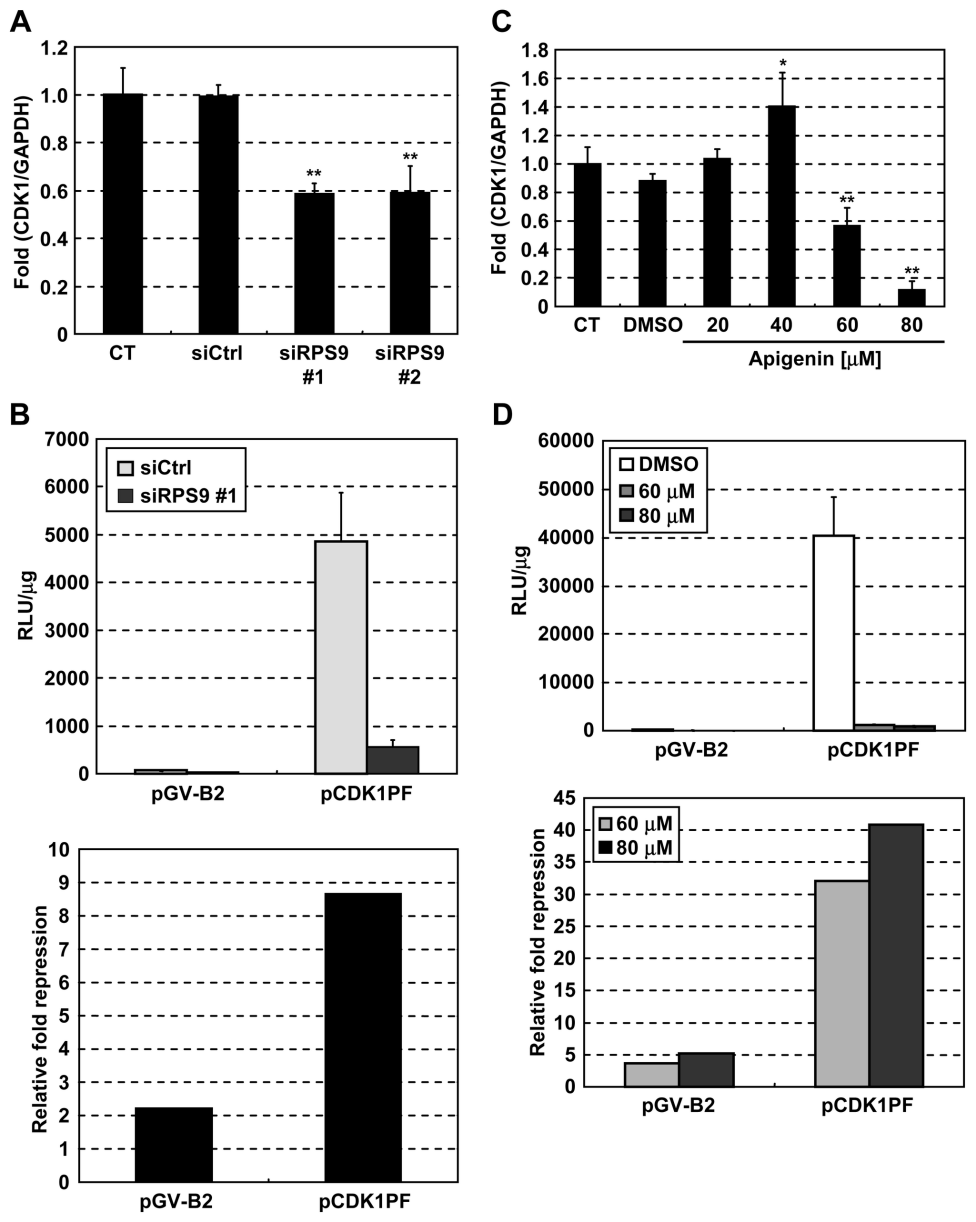
Knockdown of RPS9 as well as apigenin downregulates CDK1 mRNA at the promoter level. (A) HT-29 cells were transfected with siRPS9 or siCtrl. After 72 hr, CDK1 mRNA was quantified by real-time RT-PCR. (B) HT-29 cells were transfected with siRPS9 or siCtrl. After 48 hr, the cells were transfected with a reporter plasmid containing CDK1 promoter (pCDK1PF) or an empty plasmid (pGV-B2). After 24 hr, cell lysates were prepared for luciferase assays. (C) HT-29 cells were treated with the indicated concentrations of apigenin for 24 hr, and CDK1 mRNA was quantified by real-time RT-PCR. (D) HT-29 cells were transfected with pCDK1PF or pGV-B2. After 8 hr, the indicated concentrations of apigenin were added to the cells. After 16 hr, cell lysates were prepared for luciferase assays. CT: control, Data are means ± SD (n = 3). *P < 0.05, **P < 0.01 relative to control.

The mechanism through which knockdown of RPS9 suppressed the expression of CDK1 was investigated next. Knockdown of RPS9 inhibited the expression of CDK1 mRNA (Figure 4A). The effect of RPS9 knockdown on CDK1 promoter activity was also examined. Silencing of RPS9 suppressed CDK1 promoter activity (Figure 4B). Similarly, apigenin suppressed the expression of CDK1 mRNA (Figure 4C) and CDK1 promoter activity (Figure 4D). These results indicate that knockdown of RPS9 as well as apigenin suppresses the expression of CDK1 at the promoter level, suggesting that apigenin downregulates CDK1 by inhibiting RPS9.

### Apigenin induces G2/M arrest by binding to RPS9

**Figure 5:**
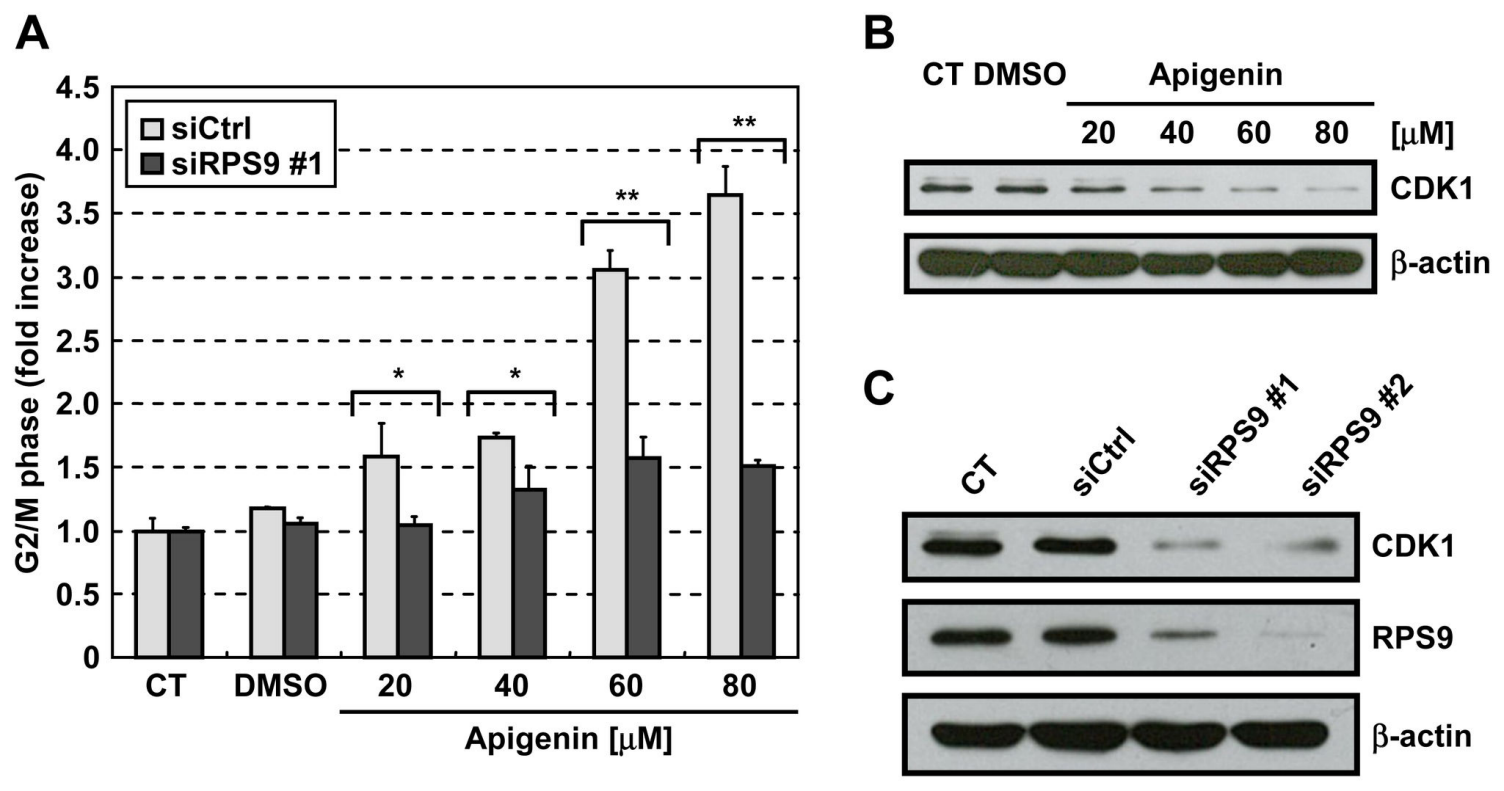
Apigenin causes cell cycle arrest at the G2/M phase by binding to RPS9. (A) HT-29 cells were transfected with siRPS9 or siCtrl. After 72 hr, the cells were treated with various concentrations of apigenin for 24 hr. Cell cycle distribution was analyzed by flow cytometry. The G2/M population in the samples without apigenin was normalized to 1. Only in this panel, comparisons were performed using unpaired Student’s *t*-test. (B) SW620 cells were treated with the indicated concentrations of apigenin. After 24 hr, the cells were lysed with RIPA buffer. The lysates were analyzed by immunoblotting. (C) SW620 cells were transfected with siRPS9 or siCtrl. After 72 hr, the cells were lysed with RIPA buffer. The lysates were analyzed by immunoblotting. CT: control, Data are means ± SD (n = 3). *P < 0.05, **P < 0.01 relative to siCtrl.

To clarify the role of RPS9 in apigenin-induced cell cycle arrest, we investigated whether knockdown of RPS9 influenced this cell cycle arrest as previously performed as to other target proteins [36,37]. Apigenin caused G2/M arrest in HT-29 cells transfected with control siRNA, but not in HT-29 cells transfected with RPS9-targeting siRNA (Figure 5A). The results show that RPS9 is required for the G2/M arrest caused by apigenin, suggesting that apigenin induces G2/M arrest by inhibiting RPS9. On the other hand, we examined whether apigenin inhibited nascent protein synthesis, since knockdown of RPS9 partially inhibited nascent protein synthesis [34]. As shown in Figure S4, apigenin decreased one of major nascent proteins indicated by an arrow with different molecular weight from that of CDK1, but not most of proteins. This reduction of nascent protein synthesis indicated by an arrow by apigenin might raise the possibility that apigenin affects protein synthesis through RPS9. We then performed the similar experiments using another human colon cancer cell line As shown in Figure 5B and C, knockdown of RPS9 as well as apigenin downregulated CDK1 in human colon cancer SW620 cells. These results suggest that apigenin downregulates CDK1 by inhibiting RPS9 not only in HT-29 cells but also in other human malignant tumor cells.

We have suggested that the flavonoid apigenin directly binds and inhibits RPS9, resulting in downregulation of CDK1 and G2/M arrest. From these results, we suppose that RPS9 enhances CDK1 expression. On the other hand, it has been reported that inhibition of RPS9 induces p53 expression, leading to tumor suppression [33]. Consistent with these results, apigenin is known to enhance p53 expression in mouse 308 keratinocytes [38], human neuroblastoma NUB-7 cells [39], and human colon cancer HCT-116 cells [27], all of which express wild-type p53. Therefore, the inhibition of RPS9 by apigenin may be one of the mechanisms through which apigenin upregulates p53. Taken together, these findings suggest that RPS9 may contribute to tumor growth by enhancing CDK1 expression and suppressing p53. A screening of other RPS9 inhibitors may therefore be useful in development of cancer therapies.

Although we clarified a mechanism by which apigenin downregulated CDK1, several other mechanisms have been reported regarding the growth inhibition induced by apigenin. As shown in Figure 1C, apigenin upregulated p21 and downregulated cyclin B1 in a p53-independent manner, but this regulation was not explained by RPS9 (Figure 3D). Since G2/M arrest is also induced by p21 upregulation and cyclin B1 downregulation, we suppose that the G2/M arrest by depletion of RPS9 (Figure 3C) was weaker than that by apigenin (Figure 1B). Moreover, apigenin is known to inhibit the NF-κB pathway [22]. Therefore, other binding proteins of apigenin except RPS9 (Figure 2A) might explain the mechanisms described above.

In the present study, we first developed a method identifying the direct targets of flavonoids (Figure S1). Since flavonoids have a variety of beneficial bioactivities such as antitumor and antiinflammatory effects [40], identification of the binding proteins of flavonoids enables us to elucidate the molecular mechanisms underlying these bioactivities of flavonoids. In fact, we clarified that RPS9 regulated the expression of CDK1 and the cell cycle by identifying apigenin-binding proteins. Further elucidation of the binding proteins of other flavonoids using our method may lead to the discovery of novel molecular mechanisms.

Most recently, the binding proteins of apigenin have been identified using a phage display cDNA library and second generation sequencing [41]. However, RPS9 was not identified in the report. We suppose that the peptides of RPS9 in the phage display library did not have the proper conformation and posttranslational modifications, and that these peptides were not identified as apigenin-binding peptides.

In conclusion, the present study suggests that the flavonoid apigenin induces G2/M arrest by directly binding and inhibiting RPS9 which enhances CDK1 expression. Our method identifying the direct targets of flavonoids should contribute to clarification of novel mechanisms regulating the growth of malignant tumor cells.

## Supporting Information

Figure S1 **Scheme for the fixation of apigenin onto magnetic FG beads with epoxy linkers.**
(TIF)

Figure S2 **RNase A does not inhibit the binding between apigenin and RPS9.** RNase A (150 µg/ml) was added to HT-29 cell extracts. After 1 hr, the extracts were incubated with apigenin-fixed (+) or empty (-) beads. Apigenin-binding proteins were purified, and bound RPS9 was detected by immunoblotting. Inp: Whole cell extracts of HT-29 cells.
(TIF)

Figure S3 **Knockdown of ribosomal protein S6 (RPS6) downregulates CDK1.** HT-29 cells were transfected with two different siRNAs targeting human RPS6 (siRPS6 #1 and #2) or a non-targeting siRNA (siCtrl). After 48 hr, the cells were lysed with RIPA buffer. The lysates were analyzed by immunoblotting. CT: control
(TIF)

Figure S4 **The effect of apigenin on nascent protein synthesis.** HT-29 cells were treated with apigenin for 24 hr and incubated in methionine-free DMEM containing apigenin for 1 hr. The cells were then treated with L-azidohomoalanine for 1 hr. The newly synthesized proteins containing L-azidohomoalanine were biotinylated and detected by immunoblotting.
(TIF)
