# Supplementary figures and images for: The Flavonoid Apigenin Downregulates CDK1 by Directly Targeting Ribosomal Protein S9
Source: PLoS One. 2013 Aug 29;8(8):e73219. doi: 10.1371/journal.pone.0073219 (PMC3756953; doi:10.1371/journal.pone.0073219)

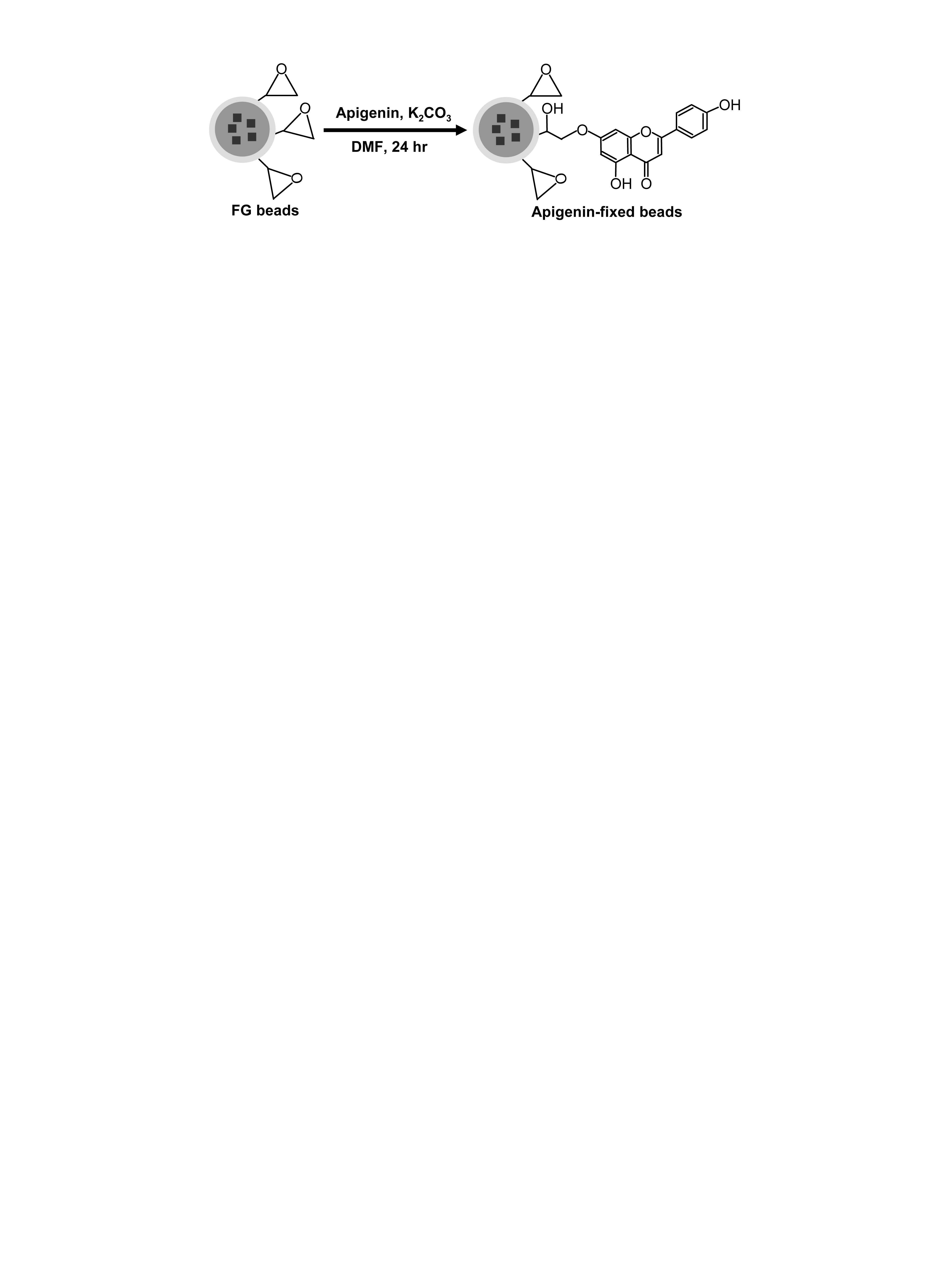

Supplement: Figure S1 — Scheme for the fixation of apigenin onto magnetic FG beads with epoxy linkers. (TIF) [file pone.0073219.s001.tif]

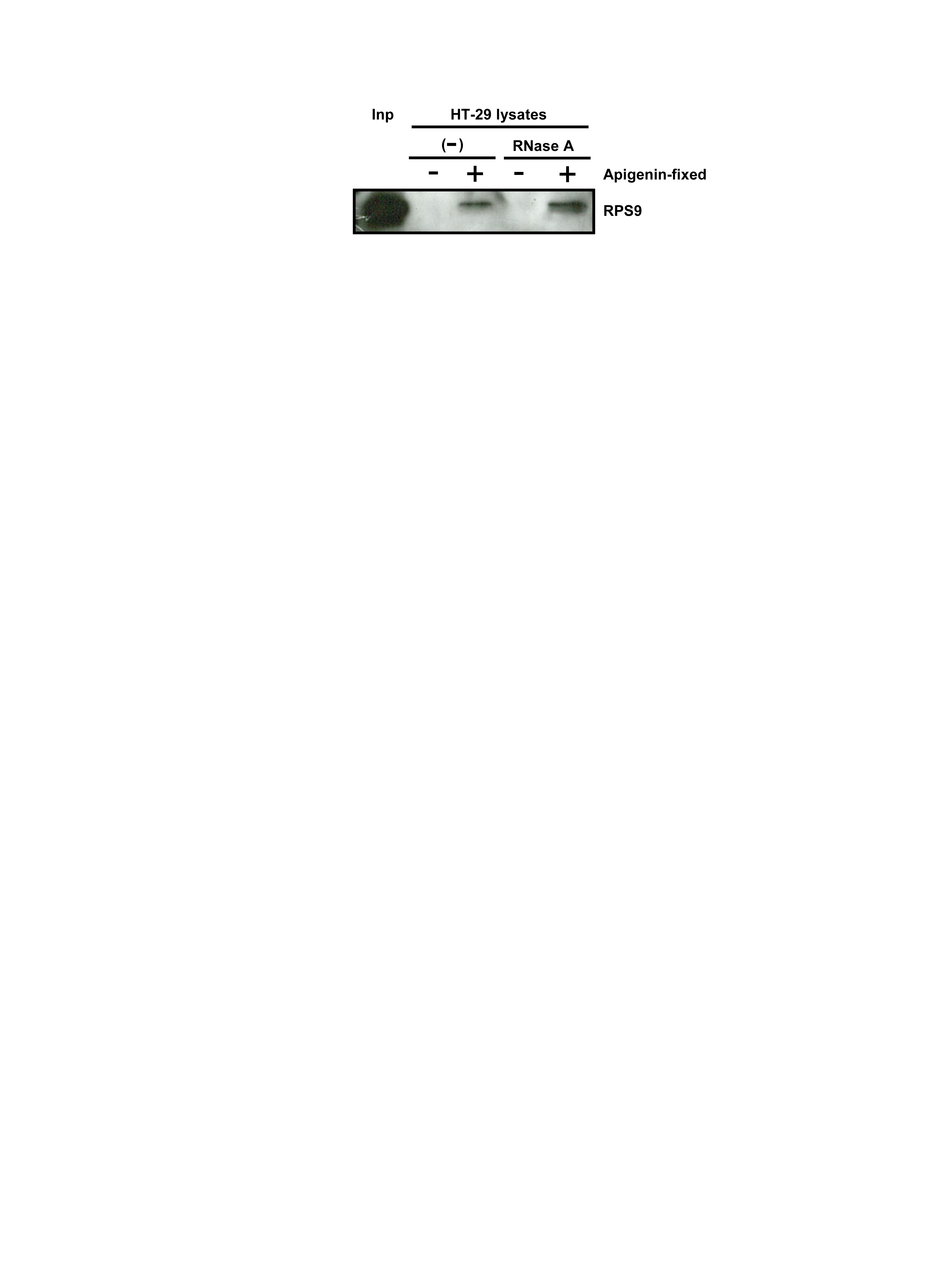

Supplement: Figure S2 — RNase A does not inhibit the binding between apigenin and RPS9. RNase A (150 µg/ml) was added to HT-29 cell extracts. After 1 hr, the extracts were incubated with apigenin-fixed (+) or empty (-) beads. Apigenin-binding proteins were purified, and bound RPS9 was detected by immunoblotting. Inp: Whole cell extracts of HT-29 cells. (TIF) [file pone.0073219.s002.tif]

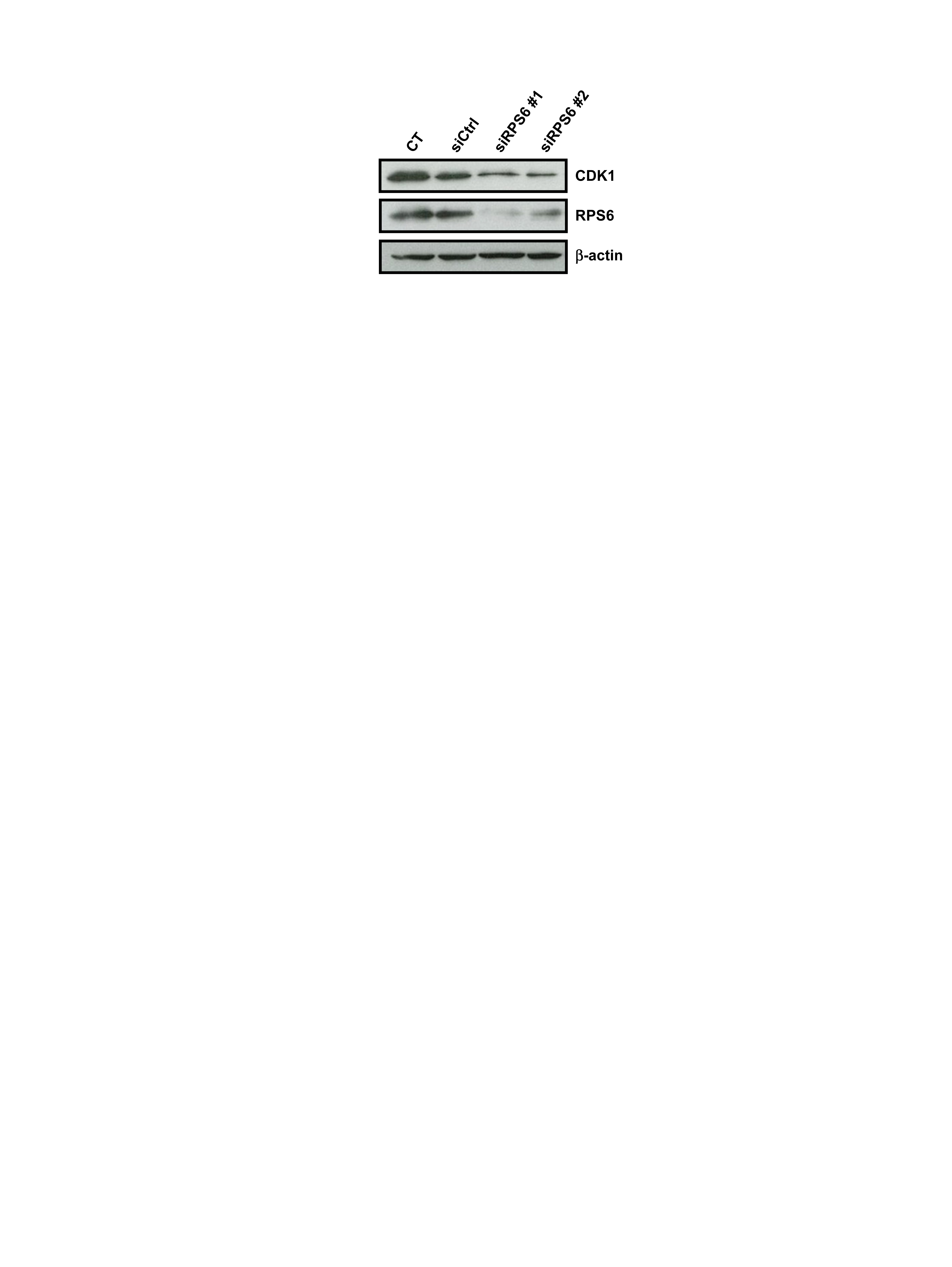

Supplement: Figure S3 — Knockdown of ribosomal protein S6 (RPS6) downregulates CDK1. HT-29 cells were transfected with two different siRNAs targeting human RPS6 (siRPS6 #1 and #2) or a non-targeting siRNA (siCtrl). After 48 hr, the cells were lysed with RIPA buffer. The lysates were analyzed by immunoblotting. CT: control (TIF) [file pone.0073219.s003.tif]

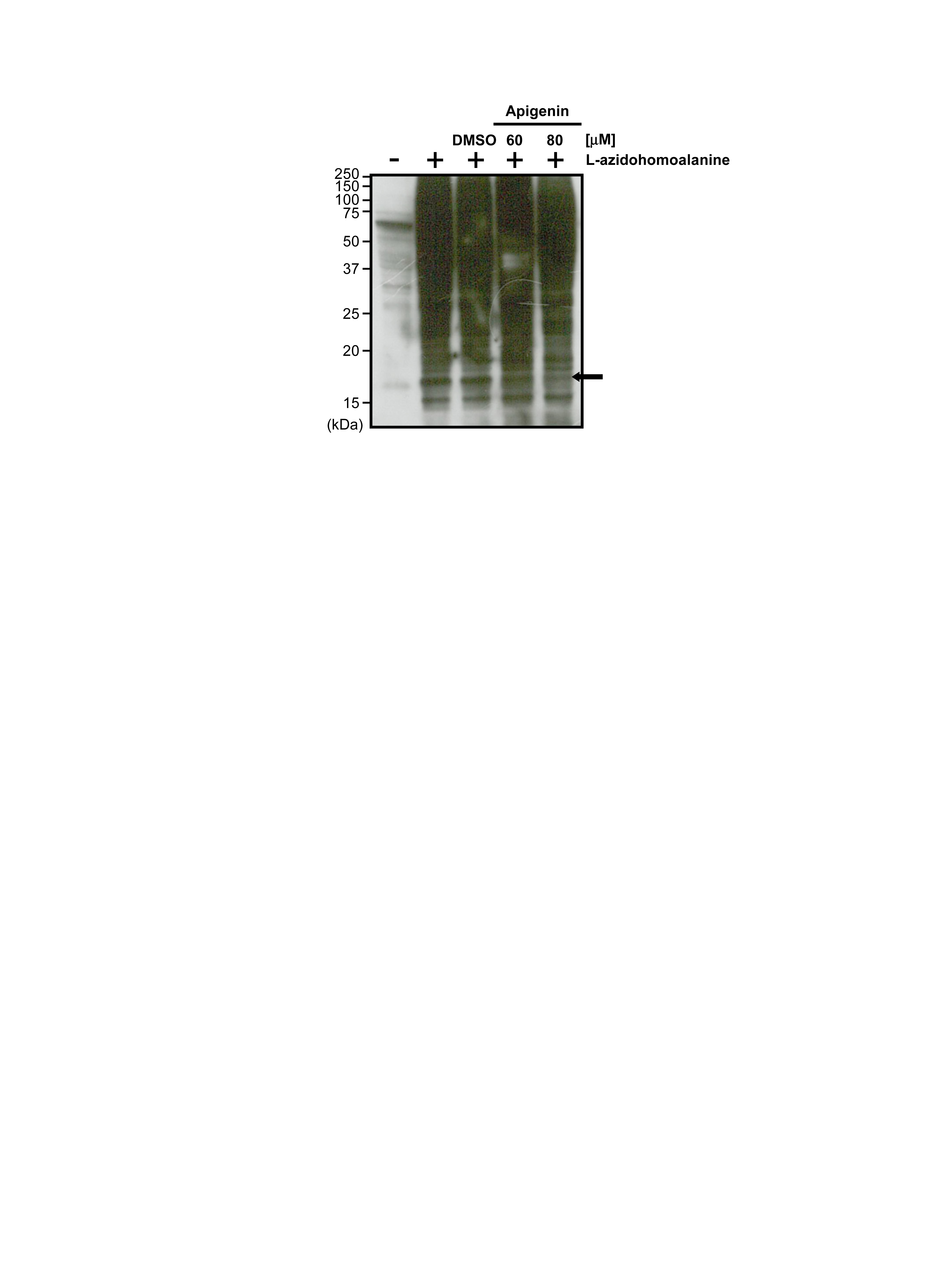

Supplement: Figure S4 — The effect of apigenin on nascent protein synthesis. HT-29 cells were treated with apigenin for 24 hr and incubated in methionine-free DMEM containing apigenin for 1 hr. The cells were then treated with L-azidohomoalanine for 1 hr. The newly synthesized proteins containing L-azidohomoalanine were biotinylated and detected by immunoblotting. (TIF) [file pone.0073219.s004.tif]
